# From Gut to Mind: Microbiota’s Impact on Schizophrenia

**DOI:** 10.1192/j.eurpsy.2025.501

**Published:** 2025-08-26

**Authors:** S. Hamzaoui, K. Mahfoudh, D. Mezri, U. Ouali, A. Aissa, R. Jomli

**Affiliations:** 1 Razi Hospital, Manouba, Tunisia

## Abstract

**Introduction:**

The human body hosts a vast array of commensal microbes known as the gut microbiota, which plays a crucial role in various physiological functions, including immune system maturation, digestion, and central nervous system development. Recent studies suggest a significant link between gut microbiota and psychiatric disorders, particularly schizophrenia. Imbalances in gut microbiota composition have been associated with symptom severity and treatment response in schizophrenia patients.

**Objectives:**

This study aims to synthesize current knowledge on the role of gut microbiota in the severity of symptoms and the prediction of treatment response in schizophrenia, highlighting its potential as a biomarker for therapeutic strategies.

**Methods:**

We conducted a systematic literature review following PRISMA guidelines, focusing on articles published between 2014 and 2024. Using databases like PubMed and Google Scholar, we searched for keywords related to gut microbiota, schizophrenia, first episode psychosis, treatment response, and symptoms severity.

**Results:**

Our review included eight studies that utilized 16S rRNA sequencing to analyze fecal microbiota. The findings revealed significant correlations between specific gut microbiota profiles and the severity of psychiatric symptoms. Notably, families such as Lachnospiraceae and Bacteroidocacae were linked to increased symptom severity, while others, including Flavobacteriaceae, Enterococcaceae and Flintibacter butyricus , correlated with symptom remission. Additionally, variations in gut microbiota composition were predictive of treatment outcomes, suggesting its role as a potential biomarker for response to antipsychotic treatments.

**Conclusions:**

The gut microbiota presents a promising avenue for understanding the complex interplay between microbial communities and psychiatric health. By identifying specific microbiota profiles associated with symptoms and treatment responses in schizophrenia, we can pave the way for novel personalized therapeutic approaches.

**Disclosure of Interest:**

None Declared

